# Neuroprotective Efficacy of Microwave‐Assisted Synthetized 2‐(5‐Cyclopropyl‐6‐Thioxo‐1,3,5‐Thiadiazinan‐3‐Yl) Acetic Acid in Mitigating Cognitive Deficits in Pentylenetetrazole‐Induced Mice Model

**DOI:** 10.1002/open.202500083

**Published:** 2025-06-17

**Authors:** Tahir Iqbal, Murad A. Mubaraki, Ahmad Khushal, Amena Arif, Fozia Fozia, Shahid Ali Shah, Ijaz Ahmad, Rasool Khan, Najeeb Ur Rehman, Ziaullah Ziaullah

**Affiliations:** ^1^ Department of Chemistry Kohat University of Science and Technology Kohat 26000 Pakistan; ^2^ Clinical Laboratory Sciences Department College of Applied Medical Sciences King Saud University Riyadh 11433 Saudi Arabia; ^3^ Medicine and surgery department Khyber Medical College Peshawar 25120 KP Pakistan; ^4^ Department of Biochemistry Federal Medical College Islamabad 44000 Pakistan; ^5^ Department of Biochemistry KMU Institute of Medical Sciences Kohat 26000 KP Pakistan; ^6^ Department of Biology The University of Haripur 22651 KPK Pakistan; ^7^ Department of Chemistry Institute of Chemical Sciences University of Peshawar 25120 Pakistan; ^8^ Natural & Medical Sciences Research Centre University of Nizwa Nizwa 616 Oman; ^9^ College of Professional Studies Northeastern University Boston 02115 MA USA

**Keywords:** acetic acid, nuclear magnetic resonance, oxidative stresses, p‐JNK, pentylenetetrazole

## Abstract

Oxidative stress is a major contributor to neurodegenerative diseases, triggering inflammation that ultimately leads to nerve cell death. This study aims to synthesize and characterize 2‐(5‐cyclopropyl‐6‐thioxo‐1,3,5‐thiadiazinan‐3‐yl) acetic acid COX‐2 and scrutinize the neuroprotective efficacy in a pentylenetetrazole‐induced mice model of epilepsy in a mice model. The compound is synthesized via microwave‐assisted method and characterized by infrared, nuclear magnetic resonance, and electron ionization mass Spectroscopy. The vivo experiments involve behavioral assessments (Morris water maze and Y‐maze tests) and biochemical analyses (antioxidant enzymes and inflammatory markers). Treatment with the synthesized compound significantly restores antioxidant enzyme levels (catalase, peroxidase peroxidation, superoxide dismutase, and glutathione), reduces lipid peroxidation, improves cognitive performance, and suppresses the expression of inflammatory proteins (p‐JNK, TNF‐α, and COX‐2). These findings suggest that 2‐(5‐cyclopropyl‐6‐thioxo‐1,3,5‐thiadiazinan‐3‐yl) acetic acid possesses strong antioxidant, anti‐inflammatory, and neuroprotective properties, making it a promising candidate for the treatment of oxidative stress‐related neurodegenerative disorders

## Graphical abstract

Microwave synthesis yielded 2‐(5‐cyclopropyl‐6‐thioxo‐1,3,5‐thiadiazinan‐3‐yl)acetic acid, confirmed by infrared, nuclear magnetic resonance, and electron ionization mass spectrometry. In a pentylenetetrazole‐induced epilepsy model, it countered oxidative stress by boosting antioxidant enzymes (catalase, peroxidase, superoxide dismutase, glutathione), reducing lipid peroxidation, inflammatory markers (p‐JNK, TNF‐α, COX‐2), and improving cognitive function. This suggests neuroprotective potential for neurodegenerative diseases..

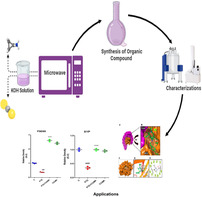

## 1 Introduction

Microwave‐assisted synthesis has revolutionized chemical synthesis by enabling significantly faster production of small molecules through microwave synthesizers than using traditional techniques. Compared to the traditional thermal heating techniques, microwave‐assisted synthesis has several benefits, such as faster reaction rates, higher product yields, better selectivity, and lower energy consumption. Microwave irradiation guarantees uniform volumetric heating, reducing side reactions and decomposition in contrast to conventional heating, which depends on conduction and convection to produce uneven temperature gradients. Furthermore, by using less solvent and producing less waste, microwave synthesis allows for cleaner reactions, quicker purification processes, and the advancement of green chemistry principles Because of these advantages, microwave technology is now the go‐to method for producing pharmaceutical and bioactive compounds efficiently.^[^ ^1^, ^2^ ^]^ Oxidative stress plays an essential function in the physiological processes of the central nervous system, together with learning and memory capabilities. There exists a delicate equilibrium between reactive oxygen species (ROS) and antioxidants when the body functions normally. However, this balance can be disrupted by an excessive accumulation of ROS, rendering antioxidant defense less effective, consequently leading to neurodegeneration and cell demise.^[^ ^3^ ^]^ In contrast to currently available antioxidants and anti‐seizure drugs, thiadiazinan derivatives provide a special structural scaffold that combines nitrogen and sulfur atoms, which can improve membrane permeability and ROS‐scavenging properties. The compound 2‐(5‐cyclopropyl‐6‐thioxo‐1,3,5‐thiadiazinan‐3‐yl) acetic acid is distinctive in structure due to the combination of a cyclopropyl ring for rigidity and a thiadiazinan core for redox balance. Thus, 2‐(5‐cyclopropyl‐6‐thioxo‐1,3,5‐thiadiazinan‐3‐yl) acetic acid may be able to reduce the oxidative stress and cognitive deficits, as neurodegenerative ailments entail progressive and irreversible degeneration of the nervous system. These conditions comprise Parkinson's disease, epilepsy, amyotrophic lateral sclerosis, and Alzheimer's disease, typically manifesting in later stages of life. Alzheimer's disease, a chronic neurodegenerative condition, predominantly affects the elderly population, ranking as the foremost cause of dementia globally and the fourth most prevalent cause of mortality. Failure of the organism's antioxidant system to counteract oxidative stress results in cellular damage, with oxidants predominantly accumulating in mitochondria.^[^ ^4^ ^]^

Epilepsy, is a neurological disorder that holds significant societal implications characterized by recurrent seizures. The underlying mechanisms of epilepsy remain unresolved despite its profound impact, and its etiology is primarily idiopathic.^[^ ^5^ ^]^

Researchers have proposed various factors that play an active role in epilepsy, including brain damage, infections, stroke, and congenital abnormalities.^[^ ^6^ ^]^ Recent research has highlighted the involvement of oxidative stress‐induced damage in triggering epileptic seizures and contributing to epilepsy's pathogenesis. Oxidative stress results in tissue damage through cell degeneration via oxidative degradation of lipids and the generation of advanced oxidation protein products due to excessive free radical production. This process leads to an increase in malondialdehyde , resultant compounds from lipid peroxidation, and a subsequent decrease in antioxidant levels such as catalase, glutathione, and glutathione peroxidase (CAT, GSH, GSH‐PX).^[^ ^7^, ^8^, ^9^ ^]^ Numerous studies have elucidated the crucial role of antioxidants like SOD in eliminating oxygen radicals to safeguard cells.

Researchers have underscored the significance of developing safe and effective antioxidants to mitigate neurodegenerative diseases. Nitrogen heterocycles are widespread in many naturally dynamic molecules that play a prominent role in drug design.^[^ ^10^ ^]^ These heterocycles constitute vital structural components of pharmaceuticals, as evidenced by historical data, a nitrogen heterocycle is present in 59% of unique small‐molecule drugs. Accordingly, newly synthesized acetic acid derivatives have shown promise as antioxidants and therapeutic agents. Pentylenetetrazole‐induced oxidative stress was used to investigate the potential of synthesized compound as an antioxidative and neuroprotective agent in BALB/c mice. In the context, the effectiveness of the therapeutic agent was evaluated by successfully reducing memory and cognizance deficits, neuroinflammation, and PTZ‐induced ROS‐intermediated neuronal synapse reduction in the mice's brain.

Researchers have suggested that epilepsy might arise from various factors such as brain injury, infections, stroke, and congenital anomalies.^[^ ^6^ ^]^ Newer research has underscored the significance of oxidative stress‐induced harm to the brain and other organs in triggering epileptic seizures and contributing to epilepsy's development. Oxidative stress involves tissue damage caused by cell degeneration through the oxidation of lipids and the creation of advanced oxidation protein products due to an excess of free radical production. Consequently, lipid peroxidation results in the production of malondialdehyde leading to a decrease in levels of antioxidants such as CAT, GSH, GSH‐PX.^[^ ^7^, ^8^, ^9^ ^]^ Several studies have illustrated the crucial role of antioxidants like SOD in scavenging oxygen radicals to safeguard cells. Previous research has established the mechanisms of action for various antiepileptic compounds, such as GABA‐mediated inhibition and modulation of voltage‐gated ion channels in epilepsy.^[^ ^11^ ^]^ Recently, antiepileptic drugs have featured functional groups such as thioxo, amino, carbolic acid, hydroxyl, carboxyl, azo, and thio groups, either discretely or in duos of these in some cases. The findings from these studies demonstrated moderate to satisfactory efficacy of the examined compounds. Therefore, the research aimed to synthesize a compound incorporating several of these functional groups concurrently to investigate their synergistic effects. Building upon the preceding discourse, the goal of this investigation was to synthesize thiadiazine derivative compounds as probable antiepileptic agents. The synthesized compounds underwent evaluation for their antiepileptic activity in a mice model having induced epilepsy. Moreover, a compound was synthesized using microwave‐assisted techniques and then evaluated for its therapeutic efficacy against epilepsy.

In the light of other discrepancies, different thiadiazinan derivatives have been previously synthesized but the current study investigates the first‐ever report on synthesis of 2‐(5‐cyclopropyl‐6‐thioxo‐1,3,5‐thiadiazinan‐3‐yl) compound and evaluates its potential neuroprotective, antioxidant, and anti‐inflammatory properties in a PTZ‐induced mice model. Furthermore, the compound may have the ability to restore cognitive performance and modulate oxidative stress and inflammatory markers suggest a novel therapeutic application in neurodegeneration.

## 2 Results

**Scheme 1:**
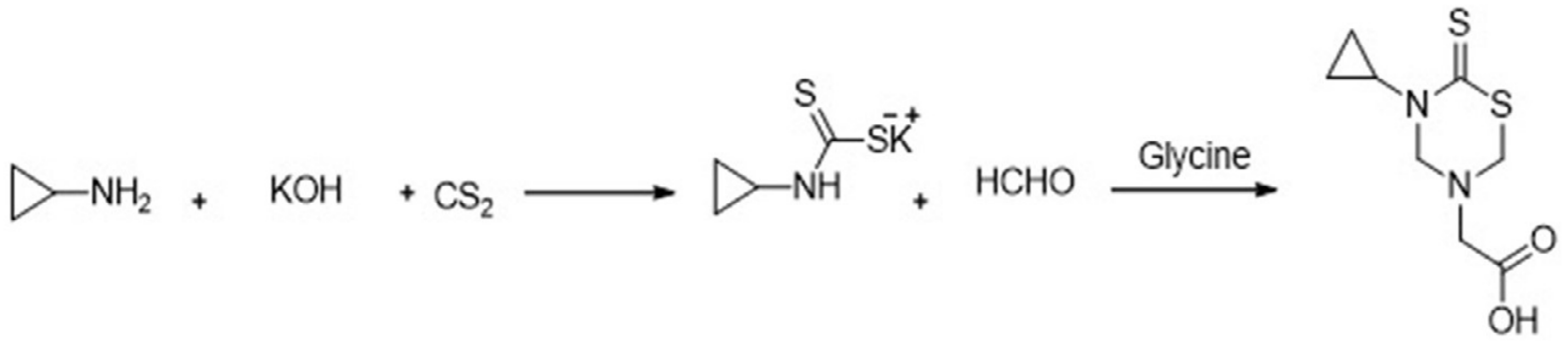
Microwave‐assisted synthesis of 2‐(5‐cyclopropyl‐6‐thioxo‐1, 3, 5‐thiadiazinan‐3‐yl) acetic acid.

The synthesis of the target compound is achieved by following **Scheme** **1**, including the carbon disulfide and primary amine reaction in an aqueous solution of potassium hydroxide. As a result, the corresponding potassium dithiocarbonate salts were formed. The addition of ethanolamine and different amino acids to phosphate buffer (pH = 7.8) following the formaldehyde treatment of these salts resulted in the cyclocondensation of the dithiocarbonate intermediate. The final products were synthesized by adjusting the pH of the reaction mixture.

### 2.1 Characterization of the Compound

Spectroscopic techniques including infrared (IR), ^1^H NMR, ^13^CNMR, and mass spectrometry were utilized to validate the arrangements of the prepared compound. The Experimental Section's spectrum results are consistent with the proposed compound structures. The compound showed accurate molecular ions in their mass spectra, with the measured MS data closely matching the calculated values. Additionally, ^1^HNMR & ^13^CNMR revealed that the concerned signals in the spectra coincided with the structure of the compound.

**Figure 1:**
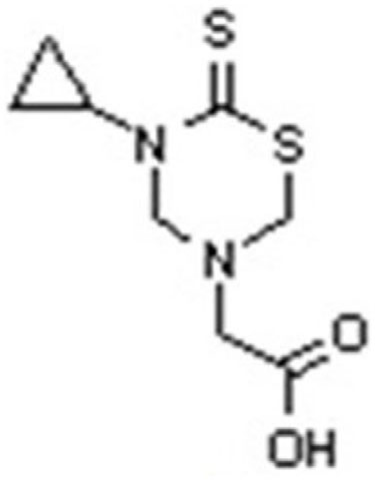
Structure of the synthesized compound.

Chemical shift patterns in ^1^H and ^13^C NMR signals were observed affirming the molecular backbone of the thiadiazine thione nucleus. In the ^1^H NMR spectra of 1,3,5‐thiadiazinethione, singlets were observed at 4.40–4.46 ppm, corresponding to protons of C‐4 and C‐6. Like this, the (C|S) thiocarbonyl carbon showed signals in the ^13^C NMR spectra that ranged from 190–192 ppm, whereas the thiadiazinane‐2‐thione nucleus's C‐4 and C‐6 signals appeared at 66–80 and 54–59 ppm, respectively. Furthermore, carboxylic carbons were evident at 171–173 ppm, as shown in **Figure** **1**.

### 2.2 Docking Studies

**Figure 2:**
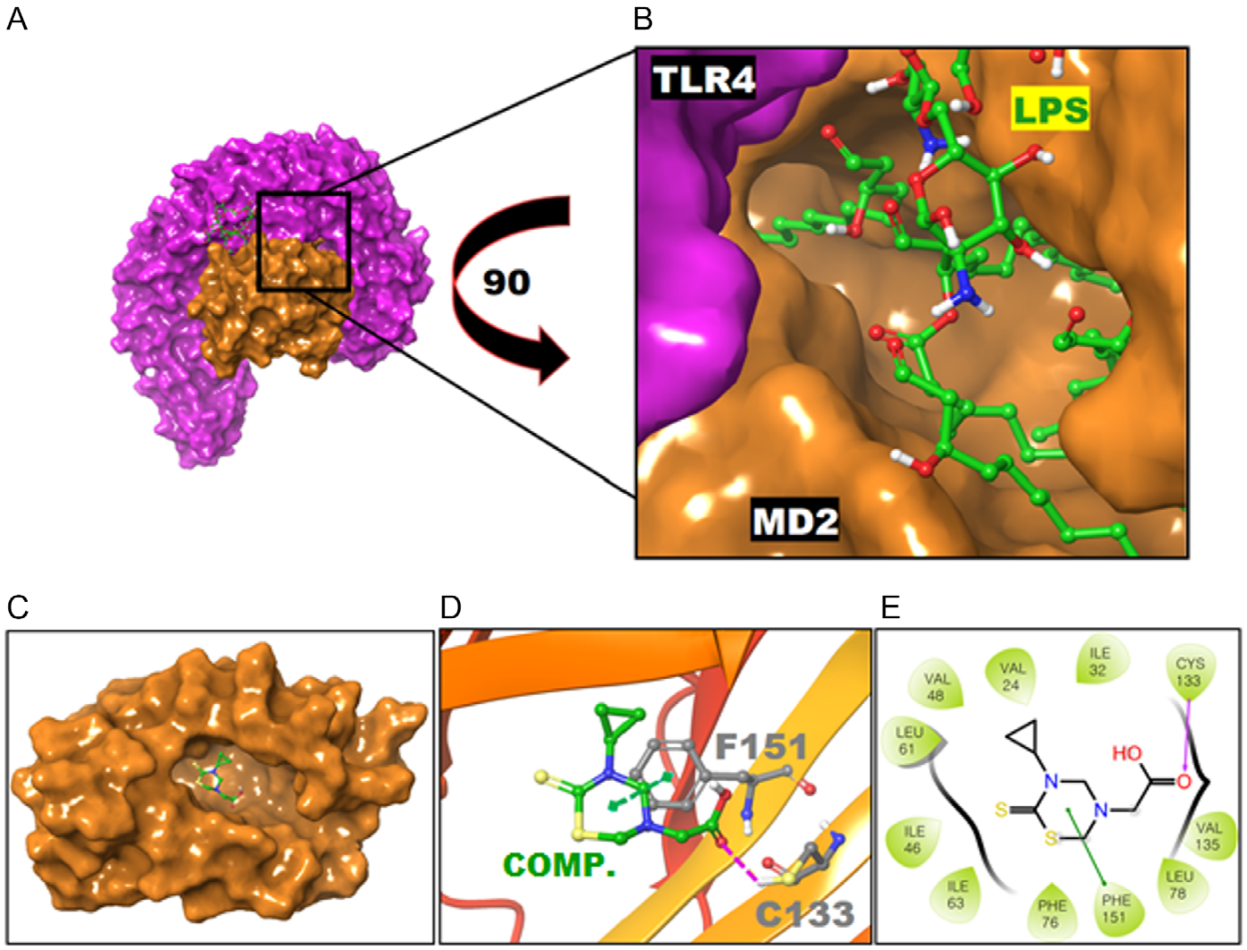
Docking of TLR4‐MD2 with synthesized compound.

The synthesized compound appeared to occupy the same site for binding and orienting the configurations to the requirements for the receiving lipopolysaccharide (LPS) molecule when it docked in the LPS binding pocket of the MD2 and TLR4 complex, which is occupied by the cocrystallized LPS. (**Figure** **2**A, B). According to our docking studies, the bulky portion of the synthesized molecule occupied the MD2 interface close to the TLR4 binding site which enables MD2 to bind TLR4 and furnishes a proper cavity‐like to lodge the LPS (Figure 2A, B), and could establish a hydrogen bond with Cys133 (Figure 2C–E).

Figure 2 demonstrates the results obtained by docking of TLR4‐MD2 with synthesized compound. (**A**) Shows the TLR4‐MD2 in complex with LPS. LPS filled the space created by the TLR4 complex and MD2. (**B**) A closer look at MD2 coupled to LPS in conjunction with TLR4. (**C**) Synthesized compound occupied the binding site of LPS in MD2 in complex with TLR4. (**D**) The MD2 and synthesized compound's 3D interaction pattern, the hydrogen bonds, and *π*–*π* stacking interaction are in magenta and green colored dashed lines. The interrelating remains of MD2 and synthesized compound are represented as the gray and green colored ball and stick models, respectively.

In addition to the synthetic molecule's inhibitory function, the docking data indicated that the compound formed a *π*‐*π* stacking contact with Phe151 of the MD2. (Figure 2D, E). Additionally, the synthesized compound's docking results in conjunction with MD2 indicated that various additional residues of MD2, which line the LPS binding cavity, developed hydrophobic contacts with the synthesized compound, as seen in Figure 2E. The synthesized compound could potentially interact with MD2, as shown by the highest docking score of −5.51 kcal mol^−1^ obtained during the docking process. The highly stable complex was produced. According to the results of the current docking studies, the most likely inhibitory effect of the produced drug is the MD2 partner of the MD2‐TLR4 complex suppression, which weakens the TLR4‐mediated signaling pathway.

### 2.3 Synthesized Compound Reduced PTZ‐Induced Oxidative Stress in Adult Mice Brain

PTZ triggers the excessive build‐up of ROS, heading to oxidative stress. In light of this, the current study aimed to synthesize the antioxidative stress activity of the synthesized compound against PTZ‐induced oxidative stress in the mouse brain. Following treatment with the synthesized compound, all brain homogenates underwent antioxidant assays, consisting of lipid peroxidation (LPO), POD, GSH, CAT, and SOD.

**Figure 3:**
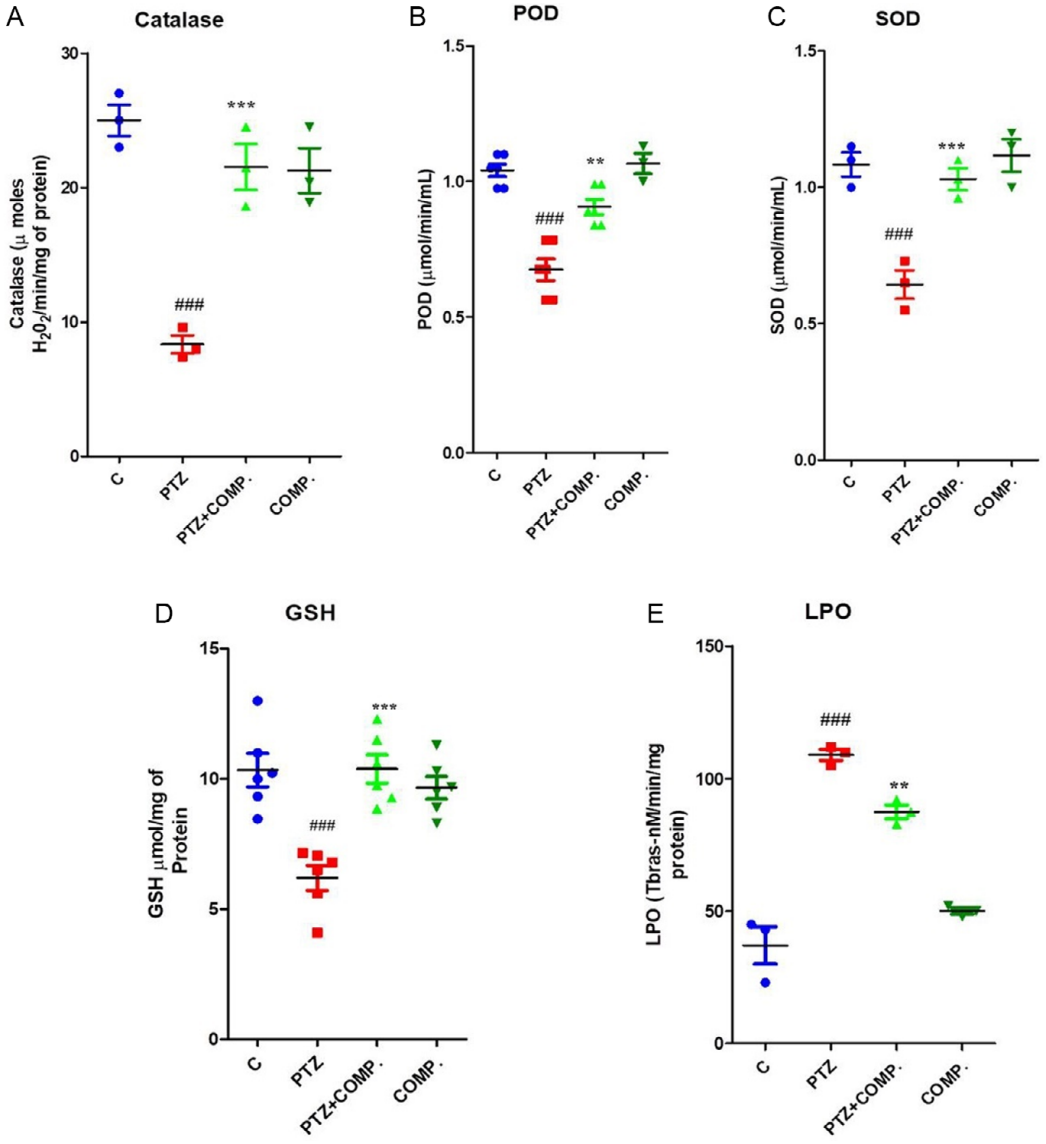
2‐(5‐cyclopropyl‐6‐thioxo‐1, 3, 5‐thiadiazinan‐3‐yl) acetic acid reduces 2‐(5‐cyclopropyl‐6‐thioxo‐1, 3, 5‐thiadiazinan‐3‐yl) acetic acid‐induced oxidative stress in adult mice.

The outcomes revealed that PTZ suppressed antioxidant enzyme activity like CAT, POD, SOD, and GSH while elevating LPO activities. Conversely, the administration of synthesized compounds notably reinstated the antioxidant enzyme activity and mitigated oxidative stress. These results imply an enhanced antioxidant potential of synthesized compounds in safeguarding the brain from PTZ‐induced oxidative harm. The depicted results are shown in Figure **3** A–E.

The graphs display the results of antioxidant enzyme assays (A) CAT, (B) POD, (C) SOD, (D) GSH, and (E) lipid peroxidase (TBARS) carried out using brain homogenates from all four groups of mice: control, PTZ‐treated, PTZ plus synthesized compound treated, and synthesized compound alone treated, respectively. The outcomes are presented as Mean ± scanning electron microscopy (SEM) of (*n* = 8) mice in every group. Significance between the PTZ‐treated and control groups is denoted as #, while * represents significance between PTZ‐treated and PTZ + 2 synthesized compound‐treated groups. The statistical significance difference is designated as **, ##*p* ≤ 0.01 and ***, ###*p* ≤ 0.001.

### 2.4 Newly Synthesized Compound Improved Pre‐ and Postsynapse against PTZ in^[^ ^8^ ^]^ Animal Model

**Figure 4:**
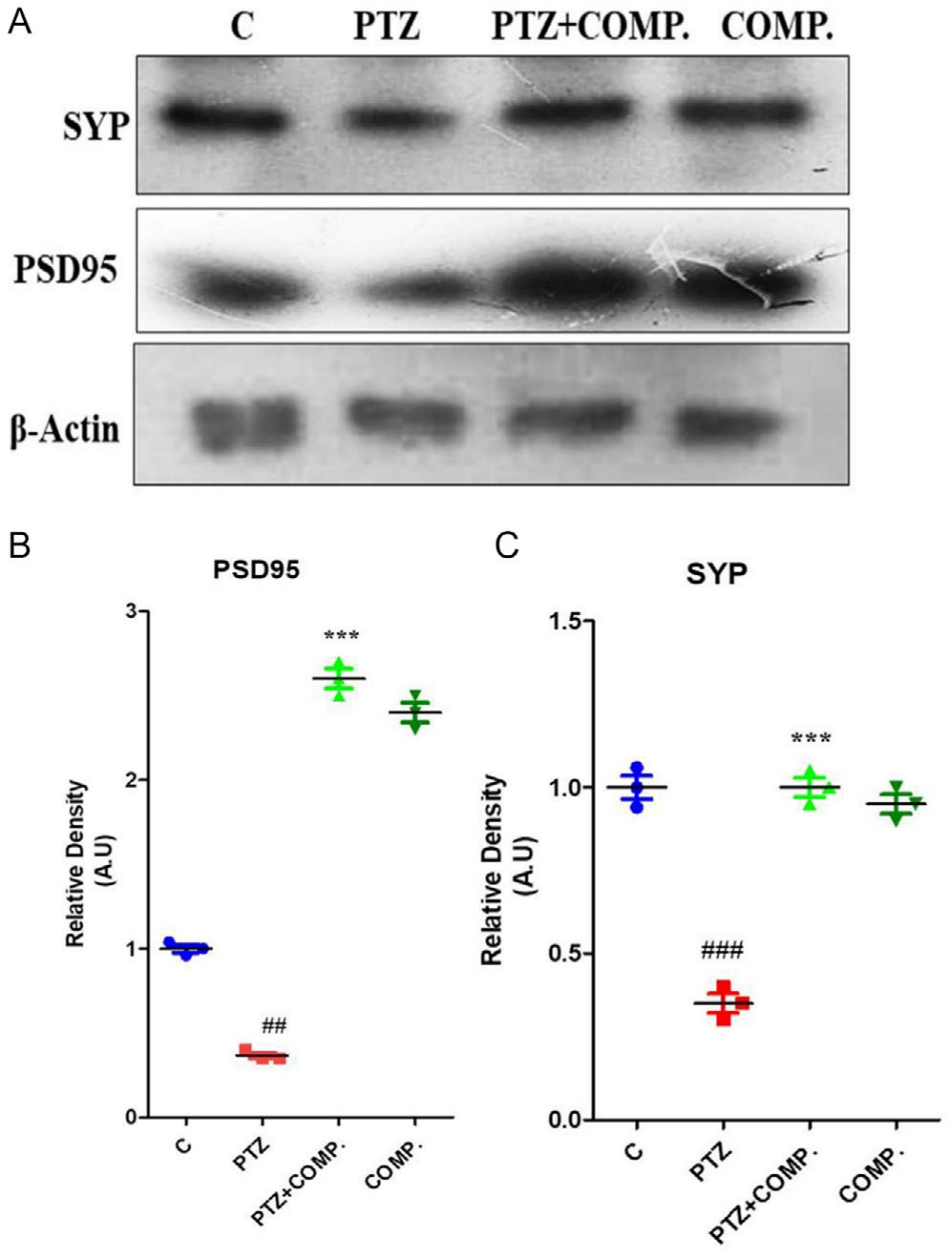
2‐(5‐cyclopropyl‐6‐thioxo‐1, 3, 5‐thiadiazinan‐3‐yl) acetic acid enhanced both pre‐ and postsynaptic protein expression against PTZ in mice.

To examine the protective effect of the synthesized compound against PTZ‐treated mice, all brain homogenates from experimental animals underwent western blot analysis. The analysis of western blotting was employed to examine the protein expression of postsynaptic density‐95 (PSD‐95), synaptophysin(SYP), and other pre‐ and postsynaptic indicators. The findings show that these proteins’ expression at pre‐ and postsynaptic locations is decreased by PTZ. On the other hand, the synthesized compound, functioning as a neuroprotective agent, brought these proteins’ expression back to nearly normal levels, as seen in (**Figure** **4** A–C).

Figure 4 shows the findings of pre‐ and postsynapse of synthesized compounds against PTZ in animal model. **(A)** The western blot results demonstrate the expression levels of PSD‐95, pre‐ and postsynaptic proteins, and SYP in the brain supernatant homogenates of experimental mice treated with PTZ alone or in conjunction with a manufactured medication. **(B, C)** The histograms display the relative densities of PSD‐95 and SYP. The Image J program was used to determine the densities of the samples. The findings are displayed as mean ± SEM in the histograms and are expressed in arbitrary units (A.U.). The statistical significance was formed at *p* ≤ 0.01 and *p* ≤ 0.001.

These findings offer insights into the impact of PTZ and its combination with synthesized compounds on the expression levels of synaptic proteins in the brain, providing valuable information on potential mechanisms underlying neuroprotective effects.

### 2.5 Synthesized Compound Improved Memory Dysfunction against PTZ in Animal Model

**Figure 5:**
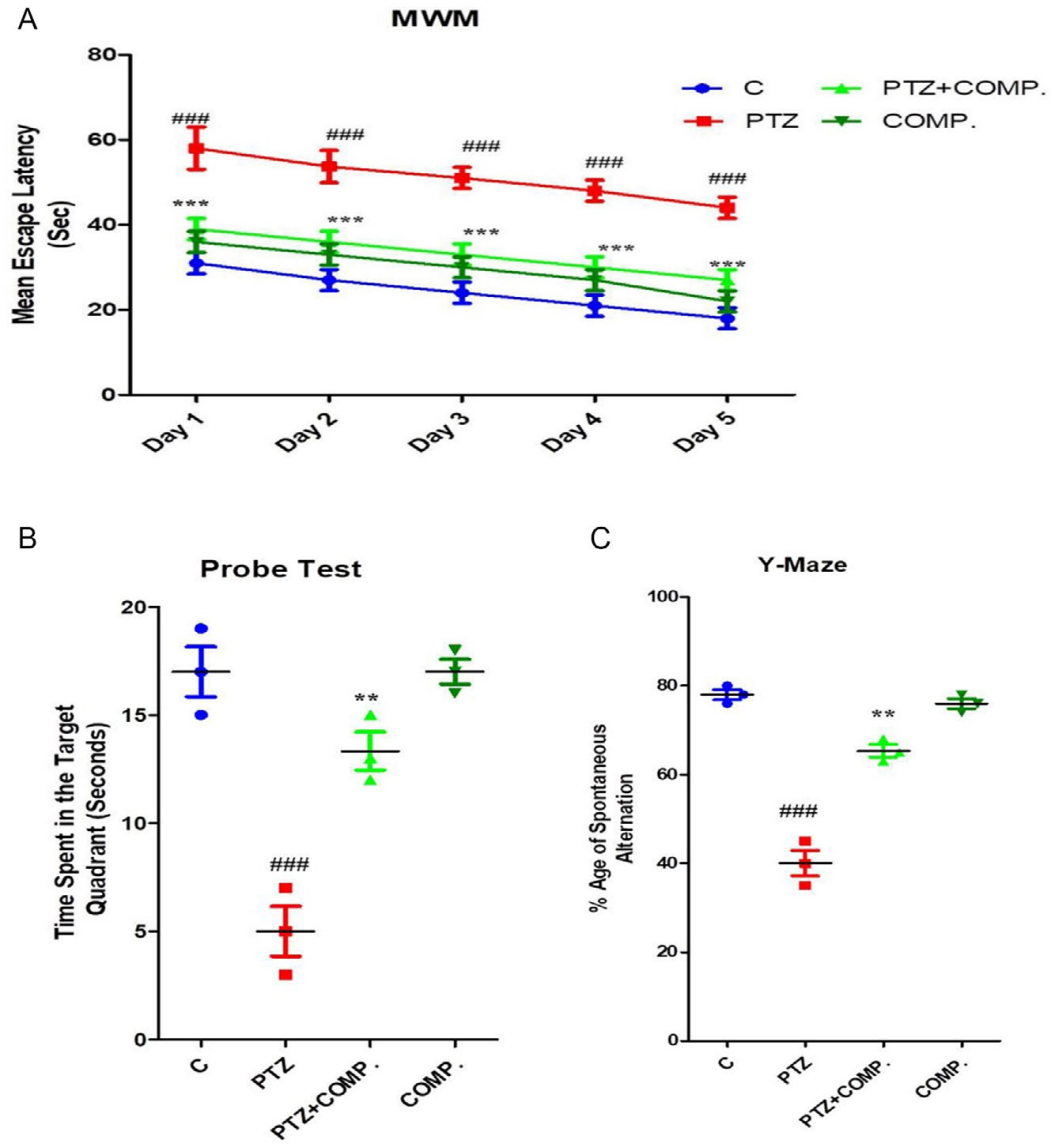
2‐(5‐cyclopropyl‐6‐thioxo‐1, 3, 5‐thiadiazinan‐3‐yl) acetic acid improved the memory in adult mice brain.

The memory impairment caused by PTZ was also lessened by synthesized compounds. The Morris water maze (MWM) and Y‐maze tests were used to assess the effect of both short‐ and long‐term memory. The mean escape delay in the MWM test was determined over a period of one to five days. In the animal model, memory impairment was indicated by the delayed average escape latency of mice treated with PTZ. In contrast to the PTZ‐treated group alone, mice treated with synthesized compound showed a significant decrease in delayed latencies to reach the platform. This improvement in escape latency indicates that PTZ‐induced memory deficits were effectively mitigated by a synthesized compound (**Figure** **5**A). PTZ‐treated mice were examined for the platform during the probe trial with a directional deficit, spending more time in nontarget quadrants than the control group. In contrast to the PTZ group, more time was spent in the target quadrant by the mice treated with the synthesized compound and exhibited directed search behavior for the platform (Figure 5B). Furthermore, the Y‐maze was used to measure short‐term memory in order to determine the percentage of spontaneous alternation. Our findings indicate that mice administered PTZ exhibited a lower percentage of spontaneous alternation, a sign of poor working memory. In contrast to the control group, mice supplied with the synthesized compound—either alone or in conjunction with PTZ showed a higher percentage of spontaneous alternation (Figure 5C). These findings imply that synthesized compound supplementation can lessen the memory deficits brought on by PTZ, enhancing the experimental mice's short‐ and long‐term memory capacities.

The outcomes of behavioral testing are reported as **(A)** Morris water maze test's average escape latency from day one to day five, **(B)** the probing test results, and **(C)** the percentage of spontaneous alteration in the Y‐maze test. The entire values are presented as mean ± S.E.M. *p* ≤ 0.01 and *p* ≤ 0.001 are significant.

### 2.6 The Synthesized Compound Inhibited p‐JNK/TNF‐α to Reduce Neuroinflammation against PTZ in Mice

**Figure 6:**
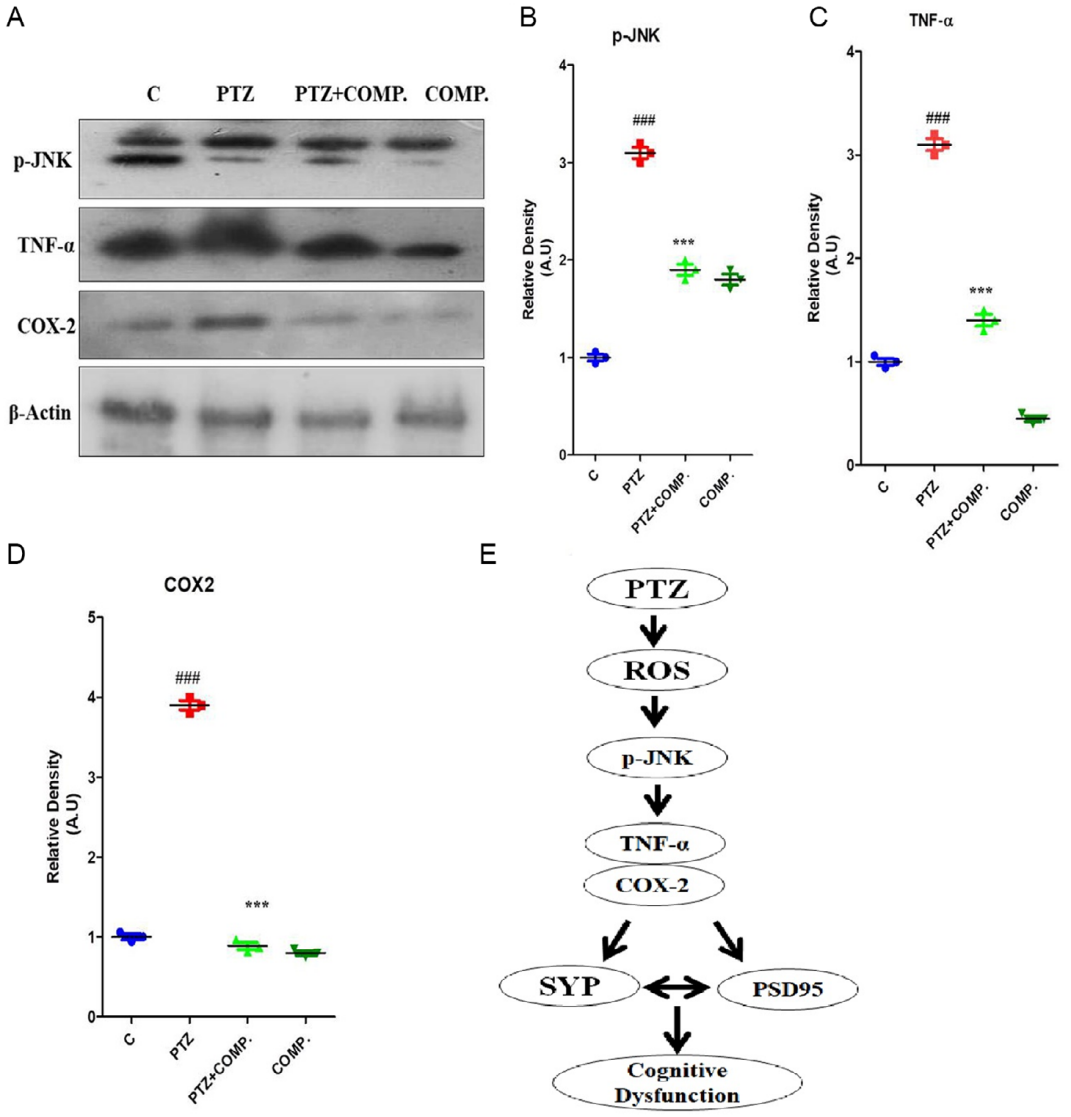
2‐(5‐cyclopropyl‐6‐thioxo‐1, 3, 5‐thiadiazinan‐3‐yl) acetic acid inhibited p‐JNK/TNF‐α to reduce neuroinflammation against PTZ in adult albino mice.

PTZ administration led to the activation of phospho‐JNK and its downstream effectors TNF‐α and COX‐2 proteins. Conversely, supplementation with HBA significantly prevented the protein expression of phospho‐JNK and its downstream TNF‐α and COX‐2 proteins, indicating its anti‐inflammatory properties (**Figure** **6**B–D). Additionally, the proposed neuroprotection pathway of the synthesized compound is illustrated in Figure 6E.

The comparison of PTZ and combined treatment with two synthesized compounds is demonstrated in **(A)** with the immunoblot of p‐JNK, TNF‐α, COX‐2, and β‐actin, and the corresponding histograms are located at **(B, C)**. The data were expressed using Image J software in A.U., and the histogram showed the mean in A.U. ± SEM. **(D)**. The synthesized compound neuroprotection signaling pathway in the PTZ animal model is displayed. The symbol * indicates PTZ versus PTZ + 2 synthesized compounds, whereas the symbol # showed the significance of control versus PTZ. ^**,^^##^ *p* ≤ 0.01 and ^***,^^###^ *p* ≤ 0.001.

## 3 Discussion

The current study was used to explore the neurotherapeutic impacts of a synthesized compound on PTZ‐induced synaptotoxicity and impaired memory in mature albino mice for the very first time. The synthesized compound was administered to male adult mice at a body weight dose of 10 mg kg^−1^. The Y‐maze and Morris water maze tests were employed to measure memory, western blotting, and antioxidant enzyme methods to assess the compound's antioxidant, anti‐neuroinflammatory, and synapse protein restoration efficacy against PTZ in male adult mice. According to our research, PTZ markedly raised antioxidant enzymes, which PTZ had repressed. This improved neural synapse function and adult male albino mice's cognitive function. Additionally, the synthesized compound notably inhibited phosphorylated JNK, TNF‐α, and COX‐2 proteins, effectively rescuing adult mice from the effects of PTZ.

Synaptic plasticity serves as a crucial foundation for learning and memory, playing a significant role in the restructuring of brain networks, particularly evident in various neurodegenerative diseases such as epilepsy. Therefore, enhancing synaptic plasticity holds particular importance in treating nervous system‐related disorders. The research confirms that treatment with synthesized compound in mice leads to increased expression of both presynaptic, such as SYP, and postsynaptic proteins like PSD‐95. A common protein in synaptic vesicles (SVs) is called SYP, and it engages in interaction with v‐SNARE SYB2. Although the exact role of SYP has long been unknown, recent experimental data points to its participation in controlling the SV membrane's SYB2 back‐trafficking and the retrieval of SYB2 from the plasma membrane following fusion.^[^ ^12^ ^]^ Mutations in the X‐chromosome‐based SYP gene have been related to frontotemporal dementia and X‐linked intellectual impairment with epilepsy.^[^ ^13^, ^14^, ^15^ ^]^ Experimental models with pathogenic variants in murine neurons have shown flawed targeting of SYB2 to SVs similar to SYP knockout neurons.^[^ ^13^ ^]^ A number of mutations cause total loss of expression and nonsense‐mediated degradation.^[^ ^15^ ^]^ SYP knockout mice serve as a valuable model for studying neurodevelopmental disorders, although their behavioral phenotype has yet to be fully characterized, with adult mice showing deficiencies mainly in object recognition and spatial learning.^[^ ^16^ ^]^ At the postsynaptic location, neurotransmitter receptors are dynamically expressed at the PSD, a complex protein network mostly present in excitatory synapses. The SHANK1, SHANK2, and SHANK3 genes encode proteins that function as multidomain scaffolds that build the PSD at excitatory synapses. SHANK proteins function as a major scaffold in the PSD by interacting with a variety of synaptic proteins.^[^ ^17^ ^]^ Cognition, a critical domain affected in NDDs, has been extensively evaluated in both pre‐ and postsynaptic models, highlighting the pivotal role of synaptic genes in regulating cognitive functions.^[^ ^18^ ^]^ In whole‐brain studies, PTZ injection has increased LPO levels in animal brains^[^ ^19^, ^20^, ^21^ ^]^ or when assessed in distinct brain regions such as the hippocampus, cortex, and striatum.^[^ ^22^, ^23^, ^24^, ^25^ ^]^ Several investigations have demonstrated that following PTZ‐induced seizures, the antioxidant enzyme activity in the rat's brain is reduced, including SOD, GPx, glutathione reductase, and CAT.^[^ ^19^, ^21^, ^26^ ^]^ The administration of acute PTZ leads to specific biochemical changes in various brain regions, with considerable changes in enzyme activity seen in the hippocampus and cerebral cortex.^[^ ^27^ ^]^ A recent study has indicated that treatment with Artemisia can mitigate PTZ‐induced oxidative stress in peripheral tissues, potentially enhancing antioxidant enzyme levels and fortifying the antioxidant defense system.^[^ ^28^ ^]^ According to these conclusions, the current study also indicates that synthesized compound not only attenuates oxidative stress by improving the functions of antioxidant enzymes such as CAT, SOD, POD, and GSH suppressed by PTZ and reducing LPO activities but also diminishes PTZ‐induced neuroinflammation in adult albino mice. Previous research has shown that by reducing oxidative stress and lipid peroxidation, natural substances like *Citrus reticulata* extracts and traditional antiepileptic medications like topiramate enhance antioxidant defenses and cognitive abilities.^[^ ^29^ ^]^ In line with these conclusions, the current study demonstrates that 2‐(5‐cyclopropyl‐6‐thioxo‐1,3,5‐thiadiazinan‐3‐yl) acetic acid significantly improved memory function, decreased lipid peroxidation, and restored antioxidant enzyme levels in PTZ‐induced mice, indicating a similar or even better neuroprotective efficacy.

PTZ has been linked to both short‐ and long‐term memory impairments, which may manifest as deficits in attention and higher order executive functions. Experimental studies in rodents have demonstrated that hippocampal CA3 region neurons are particularly susceptible to oxidative stress, leading to significant loss of neurons critical for memory function. In our current investigation, we found that treatment with synthesized compound markedly enhanced behavior and memory in albino adult mice.

The mean escape latencies of adult mice after five days of behavioral testing considerably lowered upon finding the submerged object. On the other hand, the mean escape delay decreased in the control group. The PTZ‐treated mouse group had noticeably higher escape latencies than the control group, as shown in Figure 5. This suggests that male albino mice receiving PTZ had impaired memory and problems with spatial learning. In contrast to the mouse group that received PTZ, the group that received the synthesized compound showed a notable decrease in escape latencies to the target. The phosphorylation of phopho‐JNK2 (p54 kDa) increases in animals experiencing convulsive seizures for 1–3 days, whereas phosphorylation levels remain to those in controls in kindling‐resistant mice and mice with quicker PTZ kindling development (i.e., more prone to kindling progression). JNK is implicated in numerous biological processes, including cellular differentiation, apoptosis, stress response, and neurodegeneration, as well as neuroplasticity and regeneration.^[^ ^30^, ^31^, ^32^, ^33^, ^34^ ^]^ Different responses may be exhibited by JNK isoforms, with JNK1 having higher activity while in the basal state, while JNK2 and/or JNK3 act to take part in the process of cell death.^[^ ^30^, ^33^, ^35^ ^]^ Some studies have demonstrated an increase in JNK1/2 activation in the CA1 subregion of the hippocampus following electrical kindling, showing its relationship with neuronal loss and reactive gliosis.^[^ ^35^, ^36^, ^37^ ^]^ Similarly, we observed that two synthesized compounds significantly inhibited p‐JNK, thereby reducing PTZ‐induced neuroinflammation in the brains of mice.

## 4 Conclusion

The current investigations were used to achieve 2‐(5‐cyclopropyl‐6‐thioxo‐1, 3, 5‐thiadiazinan‐3‐yl) acetic acid using microwave‐assisted methods and the synthesized compound was characterized by different techniques including IR, NMR, and electron ionization mass spectroscopy. The effectiveness of synthesized compounds was analyzed by phosphorylated JNK, TNF‐α, and COX‐2 proteins signaling abrogates PTZ‐induced oxidative stress interacted synaptotoxicity and cognitive dysfunction in mice. The results demonstrate the synthesized compound's neuroprotective ability against PTZ‐induced memory impairment and neural synapse in grown albino mice. The compound demonstrated significant antioxidant and anti‐neuroinflammatory properties, as well as the ability to restore cognitive function by modulating synaptic plasticity pathways.

## 5 Experimental Section

5.1

5.1.1

##### Chemicals

Cyclopropyle amine, potassium hydroxide, carbon disulfide, formaldehyde, glycine, pentylenetetrazol, methanol, ascorbic acid, TMS, potassium chloride, phosphate buffer saline tablets, acrylamide, ferric chloride, sulfo salicylic acid, ferric chloride, trichloroacetic acid, dextrose, thiobarbituric acid, sodium dodecyl sulfate, sodium pyrophosphate buffer, guaiacol, NADH, phenazine methosulfate, H_2_O_2_ bisacrylamide, and ammonium per sulfate were procured by Dae‐Jung Chemicals & Metals Co. Ltd and Sigma Aldrich Chemicals Company.

##### Microwave‐Assisted Synthesis of the Compound

In the study, the synthesis of the compound was carried out through microwave synthesizer named CEM Discover System (Model 908010), manufactured by CEM Company, USA, utilizing a 10 mL pressure‐rated reaction vial equipped with a magnetic stirrer. The reaction mixture initially consisted of 4 mmol of cyclopropylamine and 4 mmol of carbon disulfide dissolved in 4 mmol of aqueous potassium hydroxide (KOH). Microwave radiation was applied for 10 min at 60 W, 40 °C. After that, the irradiated mixture was mixed with formaldehyde (8 mmol), and the irradiation was carried out for 4 min under the same circumstances. After adding glycine, the mixture was microwave irradiated for an additional 5 min at 60 W and 40 °C. Cyclopropylamine, KOH, CS_2_, and formaldehyde had an overall molar ratio of 1:1:1:2 for the primary reagents. The filtered reaction mixture was ice cooled and a white precipitate formed when acidified to pH 2 with hydrochloric acid at 0–5 °C. The compound was then isolated and purified according to established protocols.^[^ ^38^ ^]^ Proton nuclear magnetic resonance, electron impact mass, and Fourier transform infrared spectrometry were carried out for characterization.

The resulting product was an amorphous white solid: 94% yield, mp of 122–123 °C. The IR spectra showed bands for O—H at 3250 cm^−1^, C=O at 1683 cm^−1^, and C=S at 1064 cm^−1^,^1^ H NMR (400 MHz, DMSO‐d6): δ 0.57–0.82 (m, 4 H, cyclopropyl ring protons), 3.28–3.37 (m, 1 H, NCH), 4.12 (s, 2 H, NCH2COOH), 4.53 (s, 2 H, NCH2S), 4.79–4.83 (s, 2 H, NCH2N). ^13^C NMR (100 MHz, DMSO‐d6): δ 10.38 (cyclopropyl ring carbons), 39.70 (NCH), 56.81 (NCH2), 57.85 (C‐6), 67.40 (C‐4), 170.69 (CO), 193.91 (CS). MS m/z: calculated for C_8_H_13_N_2_O_2_S_2_ [M]^+^ 232.3240; found 232.3830.

##### Biological Activities

Docking Studies (Methodology): Molecular docking simulation was used to predict the binding affinity of TLR4 in the complex with MD2 and the synthesized compound. A built‐in module called “Glide” in Schrodinger Maestro is dependable for molecular docking of protein‐ligand complexes.^[^ ^39^, ^40^ ^]^ The pattern identified by the experiment and retrieved from the Protein Data Bank illustrates the TLR4‐MD2 complex.^[^ ^41^, ^42^ ^]^ The preparation of the TLR4 structure complexed with MD2 before docking involved the removal of all cocrystallized unwanted heteroatoms, followed by energy minimization using the protein preparation wizard module within Schrodinger Maestro. The energy minimization utilized the OPLS4 force field along with the steepest descent algorithms and conjugate gradient. Subsequently, the grid of docking was created by choosing LPS‐bound residues of MD2, including Cys133, Lys130, Afr90, Lys122, Gly123, Phe126, Ser120, and Tyr102, using the Receptor Grid Generation protocol. The target ligand, a synthesized compound, was then prepared using the LigPrep module in Schrodinger Maestro. Finally, the docking was performed using the Glide module, with the previously prepared MD2 in complex with TLR4 structure serving as the receptor and the synthesized compound as the ligand input files. The docking protocol employed 50 conformers and generated 40 poses for the output file, utilizing standard precision docking for accuracy, while other parameters remained at default values.

Mice and their Categorization: The current investigation employed male albino mice as the experimental medium. An adult male albino mouse, which was eight weeks old, was employed for this purpose and was supplied by the Veterinary Research Institute, Peshawar. A collective of 32 animals were employed and distributed among four groups, accommodated in appropriate cages sourced from Biobase China. Before commencing experiments, mice were given sufficient time to acclimate to their new surroundings. The following is the group delineation. 1) A group of normal (*n* = 8) mice, treated with saline as a control. For behavioral and biochemical comparisons, this group acts as the positive control group. 2) A group of mice was injected with PTZ (*n* = 8) at a dose of 35 mg kg^−1^. 3) A group of mice injected with PTZ (*n* = 8) at a dose of 35 mg kg^−1^, supplemented with synthesized compound at 10 mg kg^−1^. 4) A group of mice injected with the synthesized compound (*n* = 8) (10 mg kg^−1^).

The male mice, weighing between 30–32 g, were held in a room equipped with adequate nutrition and hydration provisions. A consistent temperature of 25 °C was upheld within the compartment, and a light and dark cycle alternating every 12 h was adhered. The animals were cared for with proper attention and treatment in line with the instructions outlined via the animal ethics committee in the laboratory. Throughout the study, attentive and conscientious treatment was administered to all mice according to the guidelines set forth by the local animal ethics committee under KUST ethical certificate No. KUST/Ethical Committee/1120.

Drug Treatment: Mice in the control group were administered 0.9% saline solution via intraperitoneal injections. The PTZ group received PTZ injections on alternate days for a duration of four weeks. The PTZ + synthesized compound group initially received PTZ injections for one week, followed by PTZ and synthesized compound (10 mg kg^−1^ administered i.p.) for the subsequent three weeks. Additionally, the group treated solely with synthesized compound (10 mg kg^−1^ administered i.p.) received injections for a total of three weeks. All injections were administered intraperitoneally with utmost care.

##### Behavioral Tests

Two validated paradigms, that is, Y‐maze test for working memory and the MWM for spatial learning and memory were used in behavioral assessments to quantitatively assess cognitive function in mice. Escape latency, or the amount of time it took to find the hidden platform, was measured in the MWM for five days in a row. The percentage of spontaneous alternation in the Y‐maze was computed as a working memory indicator. For comparison, the group that received saline treatment acted as a positive control. The neuroprotective effectiveness of the synthesized compound against PTZ‐induced cognitive deficits was assessed objectively using these behavioral data.

Y‐Shape Maze Assessment: The Y‐shape maze spontaneous alternation assessment was utilized as a behavioral assessment tool for evaluating mice's inclination toward exploring unfamiliar surroundings, reflecting their spatial memory function from short to long term. The Y‐maze setup, crafted from wood, comprised three arms positioned at an angle of 120°. The dimensions of all three arms were 10 cm in width, 50 cm in length, and 20 cm in height.

Prior to the Experimental session, mice underwent a two‐day training period lasting for 10 minutes each day to acclimate them to the unfamiliar surroundings. Afterward, the mice were given four eight‐minute opportunities to freely explore the unfamiliar surroundings. Each mouse in the Y‐maze setup was placed in the center and allowed to move around freely, and their movements were visually recorded, as well as the number of times that entered each of the maze arms. Spontaneous alternation entails mice entering the three arms successively in overlapping sets of triplets. The calculation of alternation behavior utilized a formula described in prior studies.^[^ ^43^ ^]^

This test provides valuable insights into the cognitive abilities and spatial memory function of the mice under investigation.

Navigational Skills Assessment in the MWM: The assessment of spatial learning abilities in the experimental subjects was performed through the MWM. The apparatus comprised a circular water container with dimensions approximately diameter of 100 and 40 cm in height. The level of water in the MWM setup was maintained at a 26 cm depth and 23±1 °C of temperature.

Initially, the mice underwent training sessions twice daily for three repeated days. During these sessions, the mice were tasked with locating a hidden platform situated 1 cm below the water surface within one quadrant of the tank. Escape latency refers to the duration it takes for the animals to locate the platform, and was recorded for a duration of 1 min. In instances when mice failed to locate the platform within the designated time, they were manually steered to it and 10‐second stay was given there. This training regimen persisted for five days, during which the escape latency data in seconds were recorded for each experimental group.

After resting for two days, we subjected the mice to a probe test by removing the stage from the tank. During this test, mice were required to navigate the tank and locate the submerged platform. To evaluate the ability of mice to retain spatial memory, time was spent in the target quadrant, where the platform had previously been positioned.^[^ ^43^ ^]^

##### Examination of Antioxidants in Brain Homogenates

Brain homogenates were subjected to biochemical analyses after behavioral evaluations in order to measure oxidative stress markers such as lipid peroxidation (TBARS), reduced GSH,SOD, POD, and CAT. PTZ‐treated, PTZ plus compound‐treated, compound‐alone‐treated, and control groups’ enzymatic activities were quantitatively compared.

Measurement of CAT)Activity: A substantially improved method was employed to evaluate CAT activity.^[^ ^29^ ^]^ To create a 3 mL reaction mixture (final H_2_O_2_ concentration: 5.9 mM) for this experiment, 100 mL of brain supernatant, 400 mL of H_2_O_2_, and 2,500 mL of phosphate buffer (50 mM) at 5.0 pH level were blended together. The absorbance of the reaction mixture was measured at 240 nm at 1‐minute intervals in order to monitor changes over time. A change in absorbance occurring at a rate of 0.01 units per minute was defined as one unit of CAT activity. The rate at which absorbance changed was used to calculate CAT activity. This method made it possible to evaluate the CAT enzymatic activity in the test samples.

Measurement of POD Activity: A notably altered method was used to assess the POD activity.^[^ ^29^ ^]^ The reaction mixture (final guaiacol concentration: 20 mM) in POD assay was prepared in this assay by combining 100 μL of guaiacol, 300 μL of H_2_O_2_ (40 mM), 2,500 μL of phosphate buffer (50 mM, pH 5.0), and 1,000 μL of brain homogenate supernatant. The absorbance of the reaction mixture was measured at 470 nm periodically to track the changes over time.

The rate of change in absorbance was used to quantify POD activity with 0.01‐unit change in absorbance per minute is equivalent to one unit of POD activity. This modified approach enabled the assessment of POD enzymatic activity in the experimental samples.

SOD Assessment: The SOD experiment was performed with minor adjustments to the previously reported protocol.^[^ ^44^ ^]^ A reaction mixture was prepared by mixing 100 μL of phenazine methosulfate, 1,200 μL of sodium pyrophosphate buffer (0.052 mM; pH 7.0), and 300 μL of brain homogenate supernatant. After one minute, the reaction mixture was mixed with 200 μL of NADH (780 M) to initiate the enzyme reaction. Next, glacial acetic acid in the volume of 1,000 μL was added as a stopping agent. The absorbance of the reaction mixture was measured at 560 nm, and the results were protein counts, which were calculated in milligrams (mg). This made it possible to calculate the quantity of chromogen produced, which made it easier to measure the SOD activity in the test samples.

(GSH Assessment: In this analysis, the mixture used to precipitate the proteins in 1,000 μL of brain homogenate was supplemented with an equivalent volume of sulfo salicylic acid solution (4%) in order to measure the levels of reduced GSH.^[^ ^45^ ^]^ The reaction mixture was incubated for an additional hour at 4 °C before being centrifuged at 1,200 × g for 20 min in a cooled centrifuge at 4 °C. The mixture for the reaction included 2,700 μL of phosphate buffer (pH 7.2) and 200 μL of 100 mM DTNB (5,5'‐dithiobis‐(2‐nitrobenzoic acid)). The absorbance of the reaction mixture was immediately measured at 412 nm using a spectrophotometer. In milligrams per gram of tissue, GSH levels were calculated using the obtained absorbance readings.

Approximation of TBARS: The TBARS (lipid peroxidation thiobarbituric acid reactive substance) assay was conducted using a considerably modified technique.^[^ ^46^ ^]^ A reaction mixture containing 1,000 μL was formed by combining 200 μL of brain homogenate supernatant, 200 μL of 100 mM ascorbic acid, 20 μL of 100 mM ferric chloride, and 580 μL of 0.1 M phosphate buffer (pH 7.4). The reaction mixture was then accelerated by heating and stirring for an hour in a water bath at 37 °C. Then, 100 μL of a 10% trichloroacetic acid solution was required to be added to stop the reaction. Afterward, the tubes were loaded with 1,000 μL of 0.67% thiobarbituric acid and centrifuged at 2,500 × g (95 °C) for 20 min in a hot water bath. The absorbance of the supernatant at 535 nm and 37 °C was measured in a spectrophotometer to ascertain the amount of TBARS generated by each sample.

This method enabled the examination of lipid peroxidation levels within the experimental samples.

##### Western Blotting Analysis

After the treatment regimen was concluded, euthanasia was performed on all animals.^[^ ^43^ ^]^ The hippocampal brain tissue was carefully removed from the decapitated animals and immediately placed in a 1:1 ratio of phosphate buffer saline and RNA later solution on ice to maintain the tissue's integrity. After homogenizing the hippocampal brain tissue with Thermo Scientific's T‐PER solution, the supernatant was collected and refrigerated for additional analysis. The protein concentration in the supernatant was determined using the Bio‐Rad protein estimation test and absorbance measurements at 595 nm. Standardized to 30 μg per group, all protein samples were separated on gels using sodium dodecyl sulfate‐polyacrylamide gel electrophoresis at concentrations ranging from 12% to 15%. The electrophoretic run was carried out with running conditions set at 50 mA for the first 20–30 min, and then at 120 V for an hour or a half. The proteins were transferred from the gel to a PVDF membrane (Santa Cruz Biotechnology, USA) using the semidry transblot technique (Bio‐Rad) following gel electrophoresis. Monoclonal antibodies against SYP, anti‐PSD‐95, anti‐p‐JNK, anti‐actin, anti‐TNF‐α, and COX‐2 were the main antibodies employed (Santa Cruz, CA, USA). The antimouse horseradish peroxidase (Santa Cruz, CA, USA) was then coupled with the secondary antibodies. X‐ray films were responsible for the development and visualization of the results.^[^ ^17^ ^]^

##### Statistical Analysis

All X‐ray results were scanned, and then the scanned data were compiled and put through statistical analysis using computer‐based software. For this work, several programs were used, such as Image J, Prism 5 Graph Pad, and Adobe Photoshop. The results of statistical analysis showed that PTZ‐injected mice and normal saline‐treated animals had significantly different mean values (±SEM, in arbitrary units), with statistically significant differences, represented as *P* ≤ 0.05, *P* ≤ 0.01, and *P* ≤ 0.001, respectively. These statistical findings provided valuable insights into the observed differences between the experimental groups.

## Conflict of Interest

The authors declare no conflict of interest.

## Author Contributions

**Tahir Iqbal**: formal analysis (equal); funding acquisition (equal); writing—review and editing (equal). **Murad A. Mubaraki**: investigation (supporting); writing—original draft (supporting). **Ahmad Khushal**: formal analysis (supporting); writing—review and editing (equal). **Amena Arif**: formal analysis (equal); writing—review and editing ( supportive). **Fozia Fozia**: conceptualization (equal); project administration (equal); supervision (equal); writing—review and editing (equal). **Ijaz Ahmad**: conceptualization (equal); formal analysis (equal); investigation (equal). **Shahid Ali Shah**: formal analysis (supporting); writing—review and editing (supporting). **Rasool Khan**: formal analysis (supporting); writing—review and editing (supporting). **Najeeb Ur Rehman**: formal analysis (supporting); writing—review and editing (supporting).

## Data Availability

All the relevant data generated during the research project will be provided upon request from the corresponding author.
